# The case of Connective Tissue Growth Factor (CTGF) and the pit of misleading and improper nomenclatures

**DOI:** 10.1002/ccs3.12062

**Published:** 2024-12-19

**Authors:** Bernard Perbal

**Affiliations:** ^1^ International CCN Society Nice France

**Keywords:** CCN proteins, fibrosis, pamrevlumab, yin‐yang

## Abstract

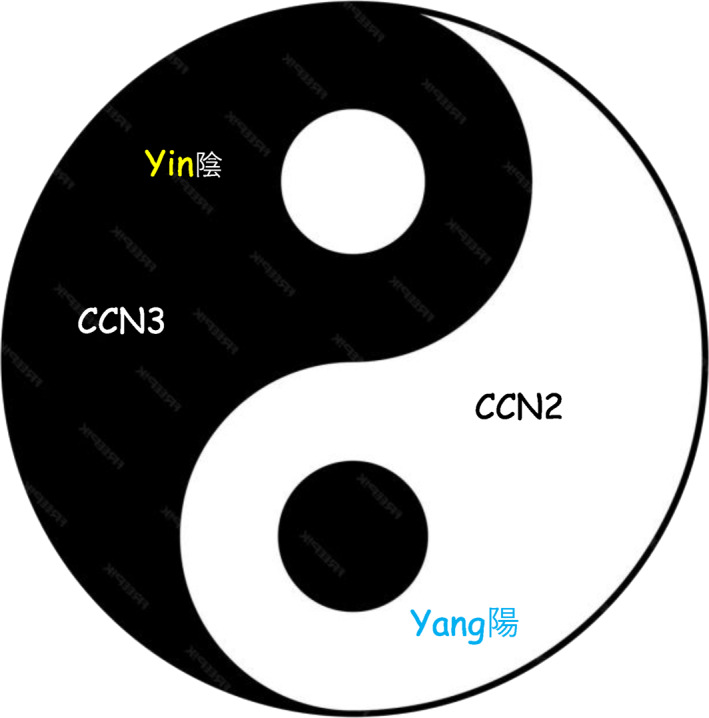

Recent developments regarding the anti‐CTGF pamrevlumab monoclonal antibody application failures led me to critically review the reasons why the International CCN Society was in favor of a unified nomenclature since 2000, and strongly supported the official HUGO Nomenclature Committee proposal to abandon original misleading or improper acronyms and renaming the CCN proteins as cellular communication network factors CCN1–6.^1^

*Foreword*: what follows is only meant to encourage researchers working in the CCN field to carefully consider the biological significance of the results that they obtain through the use of various anti‐CCN antibodies.

## 1 THE CCN FAMILY OF PROTEINS IN A FEW WORDS

Connective tissue growth factor (CTGF) belongs to a group of proteins sharing conserved organization and primary sequences. The discovery of CTGF was originally published by G. Grotendorst et al. (1991)^2^ soon after the discovery by L. Lau's laboratory of a growth‐inducible factor named CYR61 because of its rich content of cysteine residues.^3^ These two proteins are encoded by immediate early genes as defined by Almendral et al.^4^ whose expression is transcriptionally induced by cell proliferation.

The discovery of the NOV protein published in the early 1992,^5^ pointed out that in spite of its structural similarity with CTGF and CYR61, the three genes did not share a transcriptional behavior since NOV was expressed only in quiescent cells.^6^

Based on structural considerations, P. Bork^7^ proposed that these proteins constitute a new family of growth regulators. Three new members (WISP1–3), sharing the typical modular organization of the three founders, joined the CCN family of proteins.^8^

Many excellent reviews have been published regarding the central role of CCN proteins in all aspects of development, from conception to late aging and death. I will only recall here that the CCN proteins are often only considered as matricellular regulators, in spite of the growing evidence in favor of these proteins being transported to the cell nucleus where they physically interact with the RNA polymerase II complex, therefore suggesting that the CCN proteins play a role in the regulation of transcription.^9^, ^10^, ^11^

The regulatory signaling functions of the extracellular CCN proteins are connected to the control of cell behavior (proliferation, adhesion, and migration), cell growth and death (development, differentiation, and angiogenesis), and tissue repair (wound healing and regeneration). They have been directly involved in the outcome and progression of several pathologies of which cancers and fibrosis attract the attention of many biomedical professionals groups.^12^, ^13^, ^14^, ^15^, ^16^, ^17^

Interestingly, it has long been recognized that CCN1 and CCN2 can promote cancers, whereas CCN3 can suppress cancer cells growth and tumorigenesis.^10^, ^16^, ^18^, ^19^, ^20^ The conserved modular organization of CCN proteins and their interactions with a wide range of regulatory factors and ligands^21^ provides the basis for the variety of intertwined biological functions of the CCN proteins.^22^, ^23^

## 2 THE POWER OF BUSINESS

Acronyms play considerable functions. In particular, they are supposed to help communication and make it more efficient. However, the content of the signal sent by the acronym may not be received as it was meant, becoming the cause of misunderstanding. George Bernard Shaw is credited with the quote “The single biggest problem in communication is the illusion that it has taken place.” Misunderstandings abound when we make assumptions about how our communications are received by others.

Several different acronyms were used to identify CCN proteins by those who discovered them in different biological systems. Some were confusing, misleading, or inaccurate.^24^, ^25^, ^26^ Members of the International CCN Society steering committee, co‐signed (in alphabetical order) a proposal for a unifying CCN nomenclature based on the chronological order of their discovery.^27^

Indeed, the choice of NOV was not a good pick. A search of NOV on PubMed (database of the National Library of Medicine) presently gives a total of 3,668,093 results including “novel, november, sp nov”, etc. If all original acronyms should have been modified after the unified proposal mentioned above, several groups kept on using for quite a few years the original acronyms in spite of their misleading names. A strong opposition against the renaming of CTGF was orchestrated by a few groups unwilling to follow the recommendations of either the steering committee or the official HUGO Nomenclature Committee.^1^

There are several examples of laboratories being reluctant to modify the name of a gene, or a protein that they have discovered, even if they are unadapted or useless duplicates. Two main reasons are “Ego” and “fear of losing a commercial position”. It is highly probable that the FibroGen company kept on using the CTGF acronym for commercial reasons, and that the late Gary Grotendorst would not have liked to see his discovery being “denatured” by a CCN renaming. Those who were depending upon FibroGen, to get antibodies or purchase shares, might not have been keen to adopt an acronym that did not have the favors of the company.

## 3 The CCN YIN‐YANG

The first evidence in favor of yin–yang functional interactions occurring between CCN proteins was obtained along a collaborative project that we developed with the group of J. Floege.^28^ We could establish that CCN3 expression negatively correlated with growth arrest of mesangial cells and the expression of the cell cycle inhibitor P27. In contrast, CCN2 expression was found to be strongly upregulated in mesangial proliferative lesions and areas of periglomerular fibrosis. Further studies performed on mesangial cells confirmed that CCN3 downregulated CCN2 production, thereby interfering and avoiding the CCN2‐induced extracellular matrix accumulation.

A second example of yin–yang behavior of CCN2 and CCN3 was uncovered during studies of CCN proteins expression during skeletal development that were conducted with the group of M. Takigawa.^29^, ^30^ Briefly, the chondrocytic proliferation in articular, auricular, and growth plate cartilage is repressed by CCN3 (Yin), whereas it is stimulated by CCN2 (Yang). During the endochondral ossification process in the growth plate, the mature chondrocytes produce CCN2 in the hypertrophic zone to support the bone growth from inside. As shown in the case of mesangial cells, the CCN3 protein produced by resting chondrocytes maintain cell quiescence, and the cartilaginous extracellular matrix becomes calcified bone (for a detailed description of the CCN2/CCN3 yin/yang modes of action, see Kubota et al.^31^, ^32^). Chondrocytes from knock‐out CCN2 mice express 10 times more CCN3 RNA than the control wild type, whereas the levels of the other CCN RNAs were unchanged by the deletion of CCN2.

CCN2 (CTGF) expression has been associated with fibrotic disease for many years.^33^, ^34^, ^35^, ^36^, ^37^ The first results demonstrating that CCN3 inhibits the CCN2 fibrotic effects was obtained during the collaborative study of an *in vitro* model of diabetic fibrosis that we developed with the laboratory of B. Riser.^38^, ^39^ In this model, overexpression of CCN3 in mesangial cells markedly reduced the CCN2 activity and blocked the accumulation of extracellular matrix stimulated by TGF beta, whereas CCN3 expression reduced by TGF beta increased both CCN2 expression and matrix production. This yin/yang regulation was confirmed in the db/db mouse model of diabetic nephropathy.^39^ Interestingly, in human dermal fibroblasts, TGF beta also induces CCN1 and CCN2 and suppresses CCN3 expression.^40^ The anti‐fibrotic effects of CCN3 were reported to be under the control of the CCN family members‐controlled network.^41^

Furthermore, we have observed that in G59 glioblastoma cells stably transfected with a plasmid over expressing CCN2, low levels of CCN3 proteins were detected (Li, C. L. and Perbal B., 2002, unpublished results), and physical interactions between CCN2 and CCN3 have been demonstrated.^42^

Interestingly, CCN5 and CCN2 show opposite effects on cardiac hypertrophy and fibrosis. Although CCN2 induced cardiac hypertrophic growth the overexpression of CCN5 inhibited the CCN2‐induced hypertrophic phenotype.^43^ These observations and the carboxy‐proximal CT module swapping experiments reported in this work supported its critical role in the negative dominant biological properties of CCN5,^44^ whereas Monsen^45^ reported in a meeting presentation that the TSP1 domain of CCN5 was sufficient for several CCN5 functions.

In any case, these data support the need for considering the biological activities of CCN proteins in comprehensive approaches. We proposed several years ago that the family of proteins play collective and coordinated actions along a multi‐signaling axis, via their interactions with numerous regulatory factors in a spatiotemporal mode relying on combinatorial events.^46^, ^47^

## 4 NEGLECTING THE OTHER CCN PROTEINS SCIENTIFIC LITERATURE: THE TRAP

The consequences of ignoring the official CCN proteins nomenclature is not a wise choice.

Instead of putting more emphasis on the molecular and physiological basis of the observed antagonism of CCN3 on CCN2 action in several biological contexts, too many colleagues ignored voluntarily or not the whole realm of publications mentioning CCN2.

Considering the evidence reported above, and the key position of the CCN proteins in cell signaling and communication,^16^, ^21^, ^46^ it is quite disturbing that at the time the present editorial is written, among 5223 CTGF publications indexed on PubMed, only 112 (2.1%) of them mention CCN3 and 3084 (59%) do not cite CCN2.

These values strongly argue against the reductionist‐type of approaches used over the past decades which concentrated on the biological properties of the so called “connective tissue growth factor” (CTGF) even though none of the CCN proteins turned out to be a genuine growth factor.^47^ Focusing on the fine details of CTGF functions and structure while ignoring the world of CCN proteins and their biological interactions seem extremely surprising and even more inappropriate in the light of the powerful spatial biology technologies which have become available for several years.^21^, ^48^ Could the title of EE. Moussad and D. Brigstock^49^ early review “*Connective tissue growth factor: what's in a name?*” have been a warning prediction?

Aside from these basic scientific considerations, ignoring for any kind of practical reasoning, the functional interaction of CCN proteins, appears as a conceptual mistake that we pointed out many years ago (2016). Unfortunately it is frequently encountered in basic science dealing with CCN proteins biological functions. It becomes heresy and a trap, when it potentially endangers the outcome of studies aimed at validating the possible clinical applications of any of the CCN proteins. A situation that was recently commented on the LinkedIn network by one of our colleagues who was a fervent supporter of the clinical use of the pamrevlumab monoclonal antibody before the recent FibroGen clash became public.^50^ After publishing a very positive set of JCCS Bits and Bytes on this topic,^51^, ^52^, ^53^ Andrew Leask wrote “It is disappointing that since 2003 FibroGen has selected to not work with anyone expert in the CCN field. It is unsurprising that pamrevmulab failed”.

Of course A. Leask is right and I will only comment on the response of a laboratory head who became a collaborator of FibroGen to whom I asked what was his opinion about the CCN2 “isoforms” detected on western blots. He had enough to do with one band! Was it the right one?

Allow us to terminate this Editorial, on a positive note. It is our strongest wish, since the first CCN workshop in 2000, to help the development of bioactive reagents and fight CCN‐related diseases. We are still open to help whenever possible and requested. Not just a holiday season wish.

## CONFLICT OF INTEREST STATEMENT

The author declares no conflicts of interest.
